# Multimodal cues provide redundant information for bumblebees when the stimulus is visually salient, but facilitate red target detection in a naturalistic background

**DOI:** 10.1371/journal.pone.0184760

**Published:** 2017-09-12

**Authors:** Francismeire Jane Telles, Guadalupe Corcobado, Alejandro Trillo, Miguel A. Rodríguez-Gironés

**Affiliations:** 1 Department of Functional and Evolutionary Ecology, Estación Experimental de Zonas Áridas (EEZA-CSIC), Almería, Spain; 2 Department of Botany and Zoology, Masaryk University, Brno, Czech Republic; 3 Department of Integrative Ecology, Doñana Biological Station, Sevilla, Spain; University of California San Diego, UNITED STATES

## Abstract

Our understanding of how floral visitors integrate visual and olfactory cues when seeking food, and how background complexity affects flower detection is limited. Here, we aimed to understand the use of visual and olfactory information for bumblebees (*Bombus terrestris terrestris* L.) when seeking flowers in a visually complex background. To explore this issue, we first evaluated the effect of flower colour (red and blue), size (8, 16 and 32 mm), scent (presence or absence) and the amount of training on the foraging strategy of bumblebees (accuracy, search time and flight behaviour), considering the visual complexity of our background, to later explore whether experienced bumblebees, previously trained in the presence of scent, can recall and make use of odour information when foraging in the presence of novel visual stimuli carrying a familiar scent. Of all the variables analysed, flower colour had the strongest effect on the foraging strategy. Bumblebees searching for blue flowers were more accurate, flew faster, followed more direct paths between flowers and needed less time to find them, than bumblebees searching for red flowers. In turn, training and the presence of odour helped bees to find inconspicuous (red) flowers. When bees foraged on red flowers, search time increased with flower size; but search time was independent of flower size when bees foraged on blue flowers. Previous experience with floral scent enhances the capacity of detection of a novel colour carrying a familiar scent, probably by elemental association influencing attention.

## Introduction

Flowers usually present complex displays, either by means of multimodal (*e*. *g*. colour and odour) or multicomponent (*e*. *g*. shape, size and colour) cues [1]. The salience of a visual stimulus is a good predictor of initial responses and learning performance in bumblebees and honeybees [2]. Besides, when salient visual and olfactory signals are presented together, stimuli are learned better than with simple and unimodal cues [3]. Nonetheless, the performance of bees during the foraging activity is not solely constrained by the salience of stimuli (visual—size, colour, shape—and chemical—odour), but also by environmental complexity [4,5].

In homogeneous achromatic backgrounds, bees are assumed to easily adapt their visual system to such unchangeable backdrop conditions, with only stimulus intrinsic complexity (*e*. *g*. flower colour, size and shape) constraining the foraging activity [5–8]. When considering a more realistic scenario, environmental complexity is expected to increase the sources of noise as bees move along the landscape, leading to fluctuations in the perceived signals. Since flower detectability is influenced by the contrast produced with its background, visually noisy backgrounds and less salient target flowers can reduce the capacity of perception and discrimination of bees, consequently affecting the decision-making process [4,9,10]. In this sense, other cues as odour, may have an impact on the visitors’ preferences and foraging behaviour, sometimes forming stronger associations than with simply visual cues (for a review, see [11] and references therein).

Red flowers are common in natural communities of many plant species around the world [12–14]. Despite evidences of sensorial exclusion by means of colour [13,15,16], or by means of different floral traits synergistically working together with colour [17], we have long known that bees can explore red flowers [18]. Considering the trichromatic visual system of many bee species, with maximal sensitivity (λ_max_) at about 340 nm, 430 nm and 540 nm [19], red or reddish flowers (as perceived by humans, i.e., λ > 600 nm) can be of three types in the bee vision: UV-green (UV-reflecting red), blue-green and green (UV-absorbing red) [20]. For the two former types, bees can rely on the UV and blue signals to obtain the necessary chromatic information for detection and discrimination. At the extreme side, we have the UV-absorbing red flowers, perceived as achromatic by trichromatic bees (the sensitivity of most bees’ green photoreceptor at 645 nm is almost 200 times lower than at the maximum and drops to zero at about 650 nm [21]).

The mechanism by which bees detect and explore UV-absorbing red flowers is relatively unexplored [4,5,9,17]. In this case, learning may be the key to understand the relationship between the preference of bees in visiting what we expected to be a non-preferred colour (like *Trigona* bees constantly visiting *Malvaviscus arboreus* flowers in Brazil, unpublished data). Indeed, the foraging dynamics associated with the presence of both visual (*e*. *g*. colour and size) and chemical (odour, for instance as a byproduct of secondary compounds) cues, that can be used by bees as signals during the foraging activity and learning process, has barely been tested [22,23]. Moreover, our understanding at which level complex floral cues (even those not primarily related to attraction) result redundant or have an additive effect to floral visitors is limited [3,24]. One strategy adopted by bees under visually challenging foraging circumstances, is to adjust their flight speed in order to minimize the risk of missing the target flower [25]. The search time might also change, as well as accuracy, which is tightly related to the former [9,26]. Most studies focus only in single sensory modalities as an approach towards understanding the role of pollinator cognition on predicting foraging behaviour. But, to proper understand how bees deal with different trade-offs, the role of complex floral signals and background complexity must become part of the experimental setup.

In this study, we explored the role of complex floral signals, either multimodal or multicomponent, on the foraging behaviour of a generalist pollinator, the bumblebee (*Bombus terrestris*), when searching for conspicuous and inconspicuous colours presented in a visually complex background. Specifically, we aimed to answer the following questions: (i) How do search time and accuracy of bees change when searching for conspicuous (blue) and inconspicuous (red) flowers of different sizes, in the presence or absence of a second sensorial cue (odour)? (ii) How do bees adjust their flight behaviour, measured as the flight speed and total path length, when searching for conspicuous (blue) and inconspicuous (red) flowers of different sizes, in the presence or absence of a second sensorial cue (odour)? Indeed, because bees learn to associate odours with reward more rapidly, and with greater retention than colours and other visual cues [23], we tested (iii) how do bees respond to a change in stimulus colour (blue for bees trained with red and red for bees trained with blue), in the presence or absence of odour?

To assess the effect of flower size, colour and scent on the foraging strategy and efficiency of bumblebees in a complex visual environment, we followed bees as they searched for nectar at blue and red flowers of different sizes, in the presence and absence of olfactory cues. We expect that (i) flower detectability will be lower when chromatic contrast between flowers and background is low, as in the case of UV-absorbing red flowers, and bumblebees will (ii) respond differently (search time and accuracy) to the presence of multimodal information (colour and odour) during the detection of blue and red flowers of different sizes, and finally (iii) adjust their flight behaviour in order to achieve a balance during the foraging activity, when dealing with simple (size and colour) and complex (visual and olfactory) flowers.

## Materials and methods

### General setup and procedure

The experiment was performed between late May and early August of 2012 in an outdoor flight arena (length, width, height: 5 x 2.50 x 2.40 m), with the long axis in the east-west direction, in the Experimental Farm "La Hoya", belonging to the Estación Experimental de Zonas Áridas (EEZA/CSIC), Spain. The arena was built with wire-mesh and its roof was covered by dark green shading net. The south wall was overlaid with expanded polystyrene (EPS), to a height of 1.5 m. The EPS panel was painted with yellowish, brownish and greenish colours, and covered with ivy plants (*Hedera* sp.) to simulate a natural foraging environment (S1a Fig). In the north-west corner of the arena sat a bumblebee nesting box (30 x 20 x 25 cm), connected through a gated tunnel to a small feeding cage (38 x 42 x 40 cm), where bees could obtain 20% (weight/weight) sucrose solution from an uncoloured feeder outside experimental sessions. Bees had ad libitum access to pollen inside the nesting box. Colour-naive bumblebees flew into the flight arena only during bee selection and experimental sessions.

We attached 60 green EPS cubes (2 x 2 x 2 cm), with an Eppendorf tip inserted in the upper face, to the EPS panel. The Eppendorf tips contained 10 μl sucrose solution (60% weight/weight) in half of the cubes, and were empty in the other half. Bees could identify rewarded cubes by the presence of vertically coloured paper square 8, 16 or 32 mm in side, hereafter referred to as flower (S1b Fig). The squares could be blue (R: 0, G: 135, B: 255) or red (R: 255, G: 0, B: 0), printed with an Epson Stylus Photo R3000 (EPSON) colour printer onto Ilford Galerie, Smooth Pearl 290 gsm (grams/square meter) paper (ILFORD Imaging Switzerland GmbH). EPS cubes and Eppendorf tips were cleaned with ethanol 30% and haphazardly rearranged after each foraging bout–defined as a trip hive—flight cage—hive.

### Illumination and colour measurements

**Fig 1:**
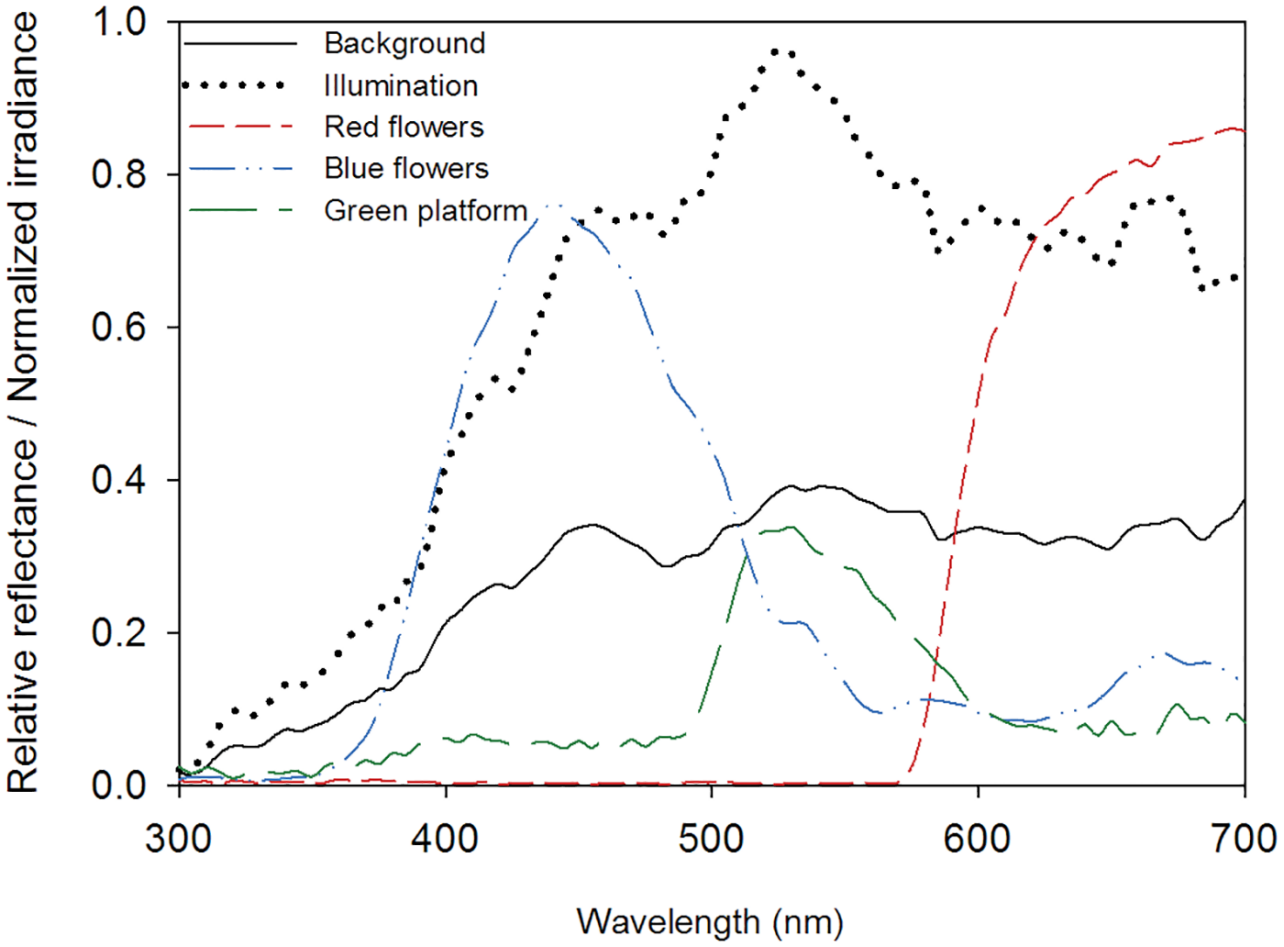
Spectral properties of stimuli, irradiance and background. Normalized irradiance and relative reflectance of background and colour stimuli within the range of 300 nm to 700 nm.

We measured illumination (vector irradiance impinging the EPS panel) and reflectance spectra of stimuli and background within the range of 300–700 nm (Fig 1) using a spectrometer (DT-MINI-2-GS Light Source, Ocean Optics USB 4000, Dunedin, FL, USA). Spectral irradiance was measure using a cosine corrector (CC-3-UV-S, Ocean Optics, Dunedin, FL, USA) coupled to the optical fibre connected to the spectrometer, after spectrometer calibration with a lamp of known output (LS-1-CAL-220, Ocean Optics). To cover the natural light variation along the day, we took five measurements of irradiance at each of three different positions within the cage at 12:00, 15:00 and 17:00 h and averaged all 45 values.

Reflectance spectra were measured relative to a white standard (WS-1 diffuse reflectance standard, Ocean Optics). For all computations, we used the normalized average of five reflectance measurements. For the red and blue colour stimuli, we used the spectral sensitivity of bumblebees [21] to compute achromatic green and brightness contrasts relative to the average background (as in [3]) and chromatic contrasts according to the colour opponent coding [27], colour hexagon [28] and receptor noise models [27–30] (S1 Table).

### Experimental procedure

We randomly assigned bumblebees to two odour treatments: unscented (UC, n = 24) and scented (SC, n = 24). Within each odour treatment, 12 bees were trained and tested with blue and the other 12 with red flowers. For the scented treatment, we added 5μl of lavender oil (*Lavandula officinalis*, from Marnys^®^, Aroma Therapy World; S2 Table for volatile organic compounds) solution (2:100 in pentane) onto rewarded EPS cubes immediately before each foraging bout. Because a highly concentrated scent could result in an aversive behaviour [23], we had previously established the concentration with a detection test, in which bees had to find rewarded EPS cubes using only the olfactory cue.

Each bumblebee experienced a single colour-odour combination, but all three flower sizes (8, 16 and 32 mm) in a pseudorandom order–each possible order was experienced by two bees for each colour-odour combination. We propose this design as an attempt to decouple the effects of stimulus size and experience.

### Bee selection and pre-training session

If the next bee had to be trained with flowers of a given treatment (UC or SC, blue or red) and starting with a given size (small, medium or large), we arranged the arena with flowers of the corresponding size and treatment and allowed five bumblebees to explore it. Once one bee started foraging, we tagged it and removed the other four. Without changing flower type or size, we allowed the tagged bee to make five foraging bouts to familiarize itself with the foraging environment. After those five foraging bouts, the experimental session started.

### Experimental session

We divided the experimental session in three rounds of ten, six and six foraging bouts. Flower size changed from round to round so that each bee experienced the three flower sizes–one size per round. During each foraging bout, we recorded the total number of visited flowers, correct (coloured rewarded platforms) and incorrect (unrewarded green platforms) choices and the time bumblebees spent flying from flower 2 to 6 (regardless of whether they were rewarded or unrewarded). We excluded the first visited flower to minimise noise: some bees flew straight from the nest to the closest point of the EPS panel, while others flew around the cage for some time before they started foraging. For each round, size and bee, we calculated the average time and divided it by the number of visited flowers (five) to obtain the “search time”–an estimate of the time bees required to find one flower. We considered a choice when a bumblebee touched the top of the platform with its front legs, regardless of whether it landed or not on the flower.

### Flight behaviour

To test whether bumblebees adapted their flight pattern to the foraging task, during the last three foraging bouts of each round we recorded bees–using a Sony video camera (DCR-SR47, Sony Hand Cam)–whenever they foraged within a framed 130 x 80 cm rectangle in the centre of the EPS panel (S1a Fig).

We developed a Matlab program (BeeTracker, available upon request) to extract from the videos the travel time (time elapsed from take-off to flower choice), total path length and average flight speed (path length divided by search time)–using the rectangular frame to convert pixels to distances. This analysis was restricted to the 1054 trajectories that did not leave the framed area: 636 for the UC treatment and 418 for the SC treatment.

Because we recorded bees with only one camera, path length and speed refer to the components of movement along the EPS panel, and ignore displacements towards or away from the camera. Because bumblebees flew within 20 cm of the EPS panel, movement along this plane provides a good approximation to 3D displacement and speed.

### Novel colour test

After the third round, we performed a novel-colour test to evaluate how bumblebees trained with blue flowers would perform when seeking nectar in red flowers and vice-versa. This test consisted of a single foraging bout, during which bees encountered 16 mm flowers of the unfamiliar colour. These flowers were scented for bees in the SC treatment, and unscented for bees in the UC treatment. We recorded the search time as in the training sessions and the number of correct and incorrect choices.

### Statistical analysis

We used generalized linear mixed models (GLMMs) with binomial distribution family and *logit* link function to examine the influence of colour, the presence or absence of odour, size or round on the proportion of correct choices. By contrast, we used linear mixed effect models (LMMs), with normal distribution, to test the effect of the same predictors over the search time, flight speed and total path length of bees. Round and size were never analysed together in a same model. Instead, all the analyses were performed twice, using either size or round. To analyse the performance of bees during the novel-colour test, we used a generalized linear model (GLM) with binomial distribution for the proportion of correct choices (*logit* link) and a linear model (LM) for the search time.

For the mixed models, we selected the most parsimonious random terms as suggested by Zuur et al. [31]. We tested all possible combinations of random terms as well as the model without random terms, and selected the model with lowest AIC value [32].

All analyses were performed using the R software, version 3.0.3 [33]. For the mixed models, we used functions GLMM and LMER, belonging to the *lme4* package [34].

## Results

### Proportion of correct choices (accuracy)

**Fig 2:**
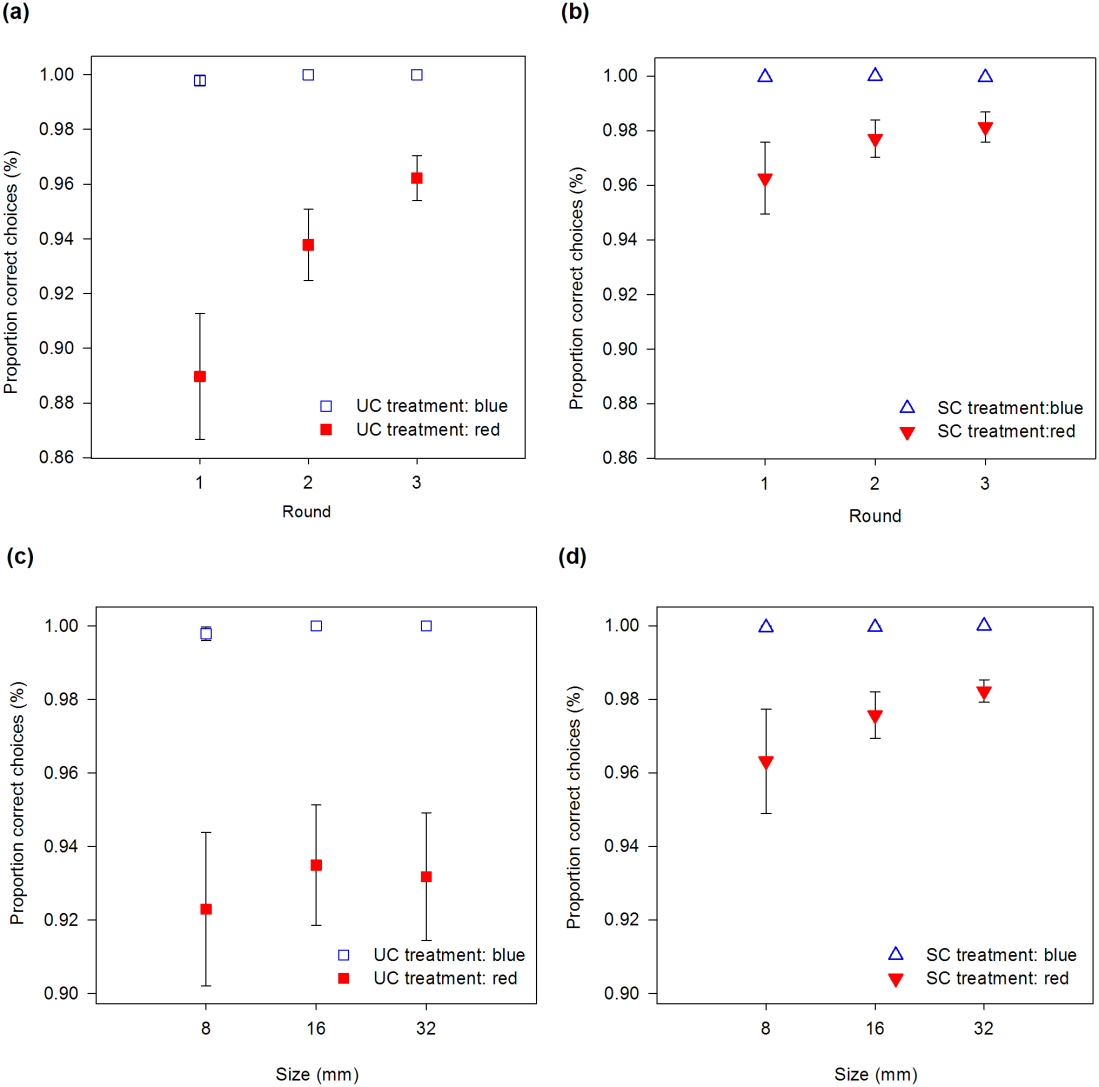
Percentage of correct choices versus round (a, b) and size (c, d) for the unscented (a, c) and scented (b, d) treatments. Error bars are standard errors.

Bees searching for blue flowers seldom landed on empty flowers (8 incorrect choices out of 11.761 choices), regardless of the odour treatment, round and size (Fig 2). Bumblebees searching for red flowers, on the other hand, started with low accuracy and their performance improved with round or in the presence of scent (Fig 2), although the positive effect of scent decreased with round (Table 1, odour treatment x round interaction). Search time also affected the proportion of correct choices: bumblebees that spent more time inspecting flowers were more accurate (Table 1).

**Table 1:** Details of the generalized linear mixed models (round and size) for the proportion of correct choices analyses.

	Model parameters		Hypothesis testing		
**Model: Round. Random term = (1|BeeID)**					
Variables	Coefficients	SE	X^2^	d.f.	P-value
Intercept	4.83	1.00			
Colour	-4.23	1.05	16.26	1	**<0.0001**
OT	1.04	0.97	1.14	1	0.28
Round	1.34	0.66	4.08	1	**0.04**
SearchTime	0.96	0.29	10.69	1	**0.001**
Colour:OT	0.66	1.05	0.40	1	0.53
Colour:Round	-0.73	0.67	1.19	1	0.27
OT:Round	-0.28	0.12	5.63	1	**0.02**
**Model: Size. Random term = (Size|BeeID)**					
Variables	Coefficients	SE	X^2^	d.f.	P-value
Intercept	5.18	1.41			
Colour	-2.22	1.47	36.63	1	**<0.0001**
OT	0.38	1.10	10.44	1	**0.001**
Size	0.21	0.13	0.70	1	0.40
SearchTime	-0.24	0.33	0.53	1	0.47
Colour:OT	0.82	1.22	0.45	1	0.50
Colour:Size	-0.20	0.13	2.59	1	0.11
OT:Size	0.003	0.02	0.02	1	0.90

In parenthesis = the most parsimonious random term. OT = odour treatment.

To understand better the odour treatment x round interaction, we analysed separately the performance of bees from the two odour treatments, using the same selected random structure for the model. In both analyses, accuracy increased with round (UC: *X*^*2*^ = 84.49, df = 1, *P* = <0.0001; SC: *X*^*2*^ = 14.09, df = 1, *P* = 0.0002), but the effect was greater for the UC (slope = 0.60; SE = 0.06) than for the SC (slope = 0.36; SE = 0.1) treatment.

In the analyses measuring the effect of flower size, standard errors were large and, as a result, only colour and odour treatment had significant effects on accuracy: bees were more accurate when searching for blue than red flowers, or when searching for scented flowers in comparison with unscented flowers. Flower size did not influence bumblebees’ accuracy (Table 1; Fig 2c and 2d).

### Search time

**Fig 3:**
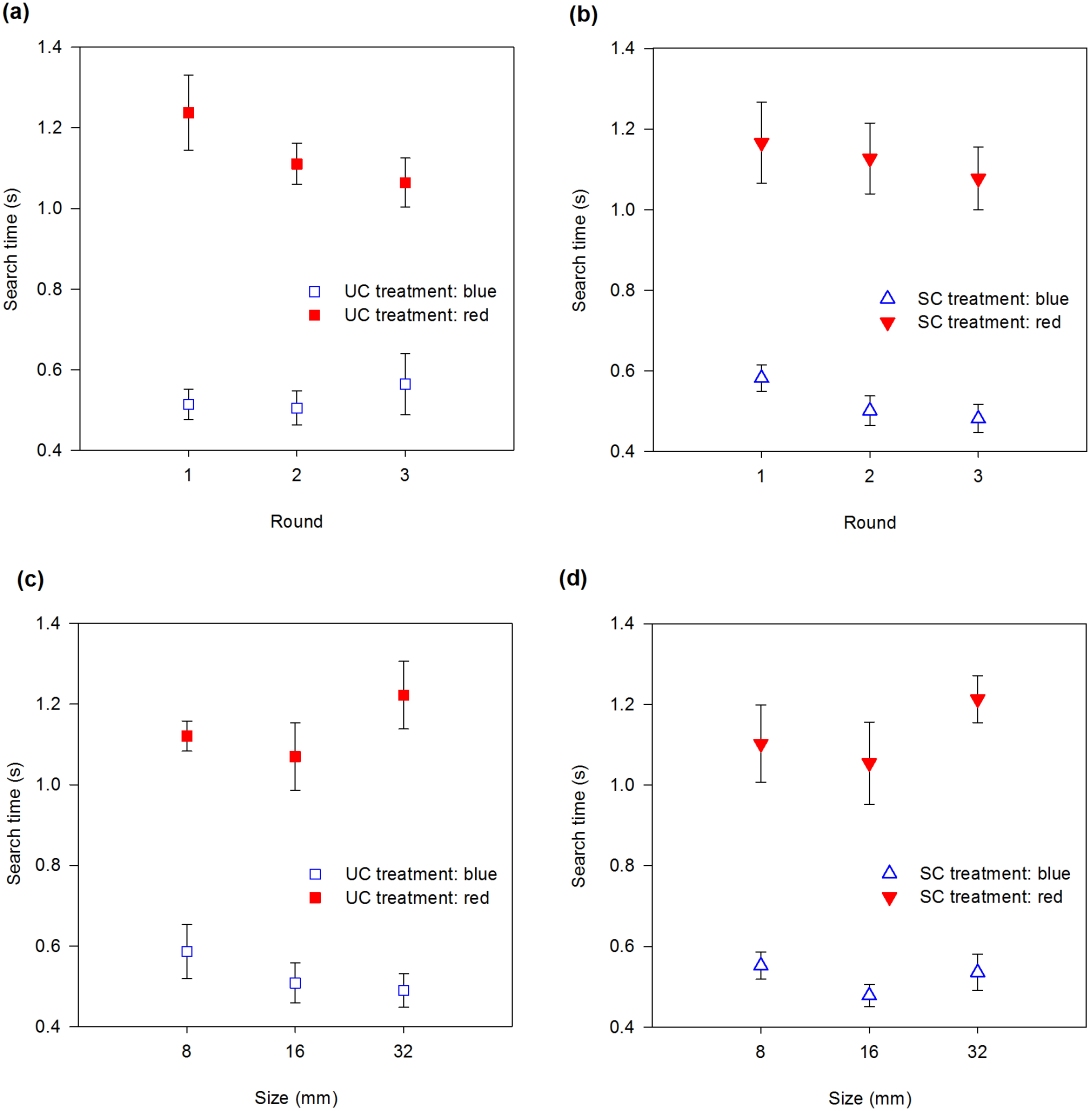
Search time (s) versus round (a, b) and size (c, d) for the unscented (a, c) and scented (b, d) treatments. Error bars are standard errors.

Search time was reduced for bumblebees searching for blue flowers in comparison with bumblebees searching for red flowers (Fig 3). For bees searching for red flowers, search time decreased with round (Table 2, Fig 3a and 3b).

**Table 2:** Details of the linear mixed models (round and size) for the search time analyses.

	Model parameters		Hypothesis testing		
**Model: Round. Random term = (1|BeeID)**					
Variables	Coefficients	SE	X^2^	d.f.	P-value
Intercept	-0.65	0.09			
Colour	0.82	0.11	205.18	1	**<0.0001**
OT	0.09	0.11	0.02	1	0.88
Round	-0.02	0.03	5.03	1	**0.02**
Colour:OT	-0.03	0.11	0.07	1	0.79
Colour:Round	-0.01	0.04	0.13	1	0.71
OT:Round	-0.04	0.04	1.18	1	0.28
**Model: Size. Random term = (1|BeeID)**					
Variables	Coefficients	SE	X^2^	d.f.	P-value
Intercept	-0.59	0.08			
Colour	0.64	0.09	42.30	1	**<0.0001**
OT	-0.06	0.09	0.38	1	0.54
Size	-0.005	0.003	3.29	1	0.07
Colour:OT	-0.03	0.11	0.07	1	0.79
Colour:Size	0.008	0.003	6.14	1	**0.01**
OT:Size	0.004	0.003	1.20	1	0.27

In parenthesis = the most parsimonious random term. OT = odour treatment.

Flower size itself did not affect search time, but its interaction with colour did (Table 2, *P* = 0.01). To study this interaction, we reanalysed colours independently. When bees were searching for red flowers, search time increased with size (slope = 0.005, SE = 0.002; *X*^*2*^ = 4.59, df = 1, *P* = 0.03). For blue flowers, in turn, the slope of the regression was slightly negative (slope = -0.002, SE = 0.001), although not statistically different from zero (*X*^*2*^ = 1.22, df = 1, *P* = 0.27).

### Average flight speed

**Fig 4:**
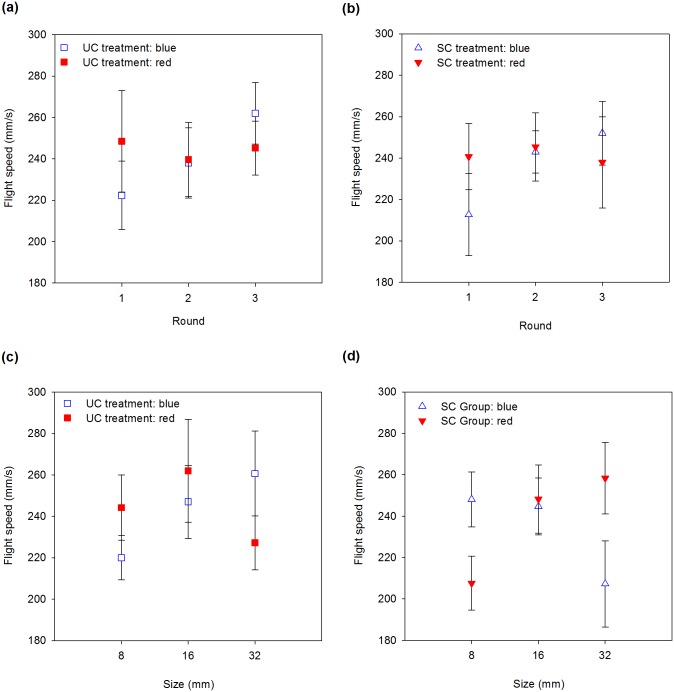
Averaged flight speed (mm/s) versus round (a, b) and size (c, d) for the unscented (a, c) and scented (b, d) treatments. Error bars are standard errors.

Round and its interaction with flower colour significantly affected average flight speed (Table 3). We therefore reanalysed the flight speed separately for each colour. Bees searching for blue flowers flew slowly during the first round, increasing their flight speed as training progressed (Fig 4a and 4b. Slope = 0.10, SE = 0.03; *X*^*2*^ = 9.20, df = 1, *P* = 0.002), while bumblebees searching for red flowers flew at the same speed throughout the experiment (*X*^*2*^ = 0.0001, df = 1, *P* = 0.99). Average flight speed was not affected by odour treatments (Fig 4a and 4b).

**Table 3:** Details of the linear mixed models (round and size) for the flight speed analyses.

	Model parameters		Hypothesis testing		
**Model: Round. Random term = (1|BeeID)**					
Variables	Coefficients	SE	X^2^	d.f.	P-value
Intercept	5.27	0.10			
Colour	0.20	0.12	3.13	1	0.08
OT	-0.02	0.11	0.05	1	0.82
Round	0.10	0.04	5.41	1	**0.02**
Colour:OT	0.03	0.09	0.10	1	0.75
Colour:Round	-0.10	0.05	4.37	1	**0.04**
OT:Round	-0.003	0.05	0.004	1	0.95
**Model: Size. Random term = (1|BeeID)**					
Variables	Coefficients	SE	X^2^	d.f.	P-value
Intercept	5.48	0.08			
Colour	-0.05	0.10	0.33	1	0.57
OT	-0.005	0.10	0.24	1	0.62
Size	-0.001	0.003	0.07	1	0.79
Colour:OT	0.04	0.09	0.20	1	0.65
Colour:Size	0.002	0.004	0.56	1	0.45
OT:Size	-0.002	0.004	0.27	1	0.60

In parenthesis = the most parsimonious random term. OT = odour treatment.

When considering flower size in the analyses, none of the parameters measured affected the average flight speed (Fig 4c and 4d. Table 3).

### Path length

**Fig 5:**
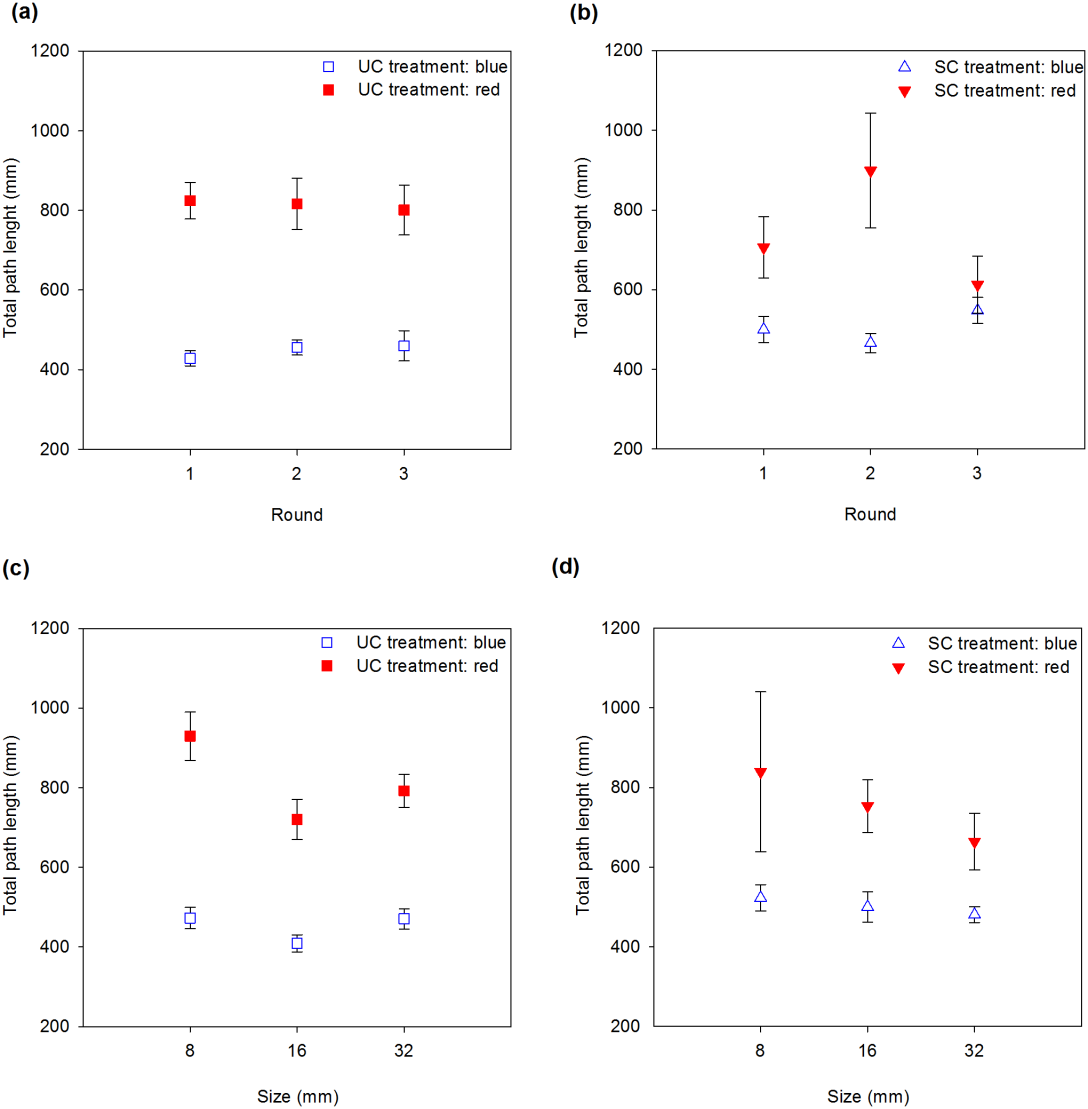
Total path length of bumblebees (mm) versus round (a, b) and size (c, d) for the unscented (a, c) and scented (b, d) treatments. Error bars are standard errors.

Regardless of whether we included round or flower size in the model, colour and its interaction with odour treatment significantly affected path length (Table 4). Path length was shorter when bees were searching for blue rather than for red flowers, although the difference decreased in the presence of scent (Fig 5).

**Table 4:** Details of the linear mixed models (round and size) for the total path length analyses.

	Model parameters		Hypothesis testing		
**Model: Round. Random term = (1|BeeID)**					
Variables	Coefficients	SE	X^2^	d.f.	P-value
Intercept	6.01	0.12			
Colour	0.74	0.14	27.94	1	**<0.0001**
OT	0.12	0.14	0.77	1	0.38
Round	0.04	0.05	0.57	1	0.45
Colour:OT	-0.25	0.11	5.78	1	**0.02**
Colour:Round	-0.07	0.06	1.67	1	0.20
OT:Round	-0.007	0.06	0.01	1	0.91
**Model: Size. Random term = (1|Size)**					
Variables	Coefficients	SE	X^2^	d.f.	P-value
Intercept	6.08	0.12			
Colour	0.67	0.11	36.46	1	**<0.0001**
OT	0.16	0.11	2.01	1	0.15
Size	0.0006	0.006	0.01	1	0.90
Colour:OT	-0.23	0.10	5.70	1	**0.02**
Colour:Size	-0.005	0.005	0.99	1	0.32
OT:Size	-0.003	0.005	0.39	1	0.53

In parenthesis = the most parsimonious random term. OT = odour treatment.

When colours were analysed separately, the presence of odour reduced path length when bumblebees were searching for red flowers (slope = -0.14, SE = 0.09), although the difference was not statistically significant (*X*^*2*^ = 1.82, df = 1, *P* = 0.09). Somewhat surprisingly, when bumblebees were searching for blue flowers, the presence of odour increased path length (slope = 0.10, SE = 0.06), but once again not statistically significantly (*X*^*2*^ = 2.74, df = 1, *P* = 0.09).

### Novel-colour test: Proportion of correct choices and search time

**Fig 6:**
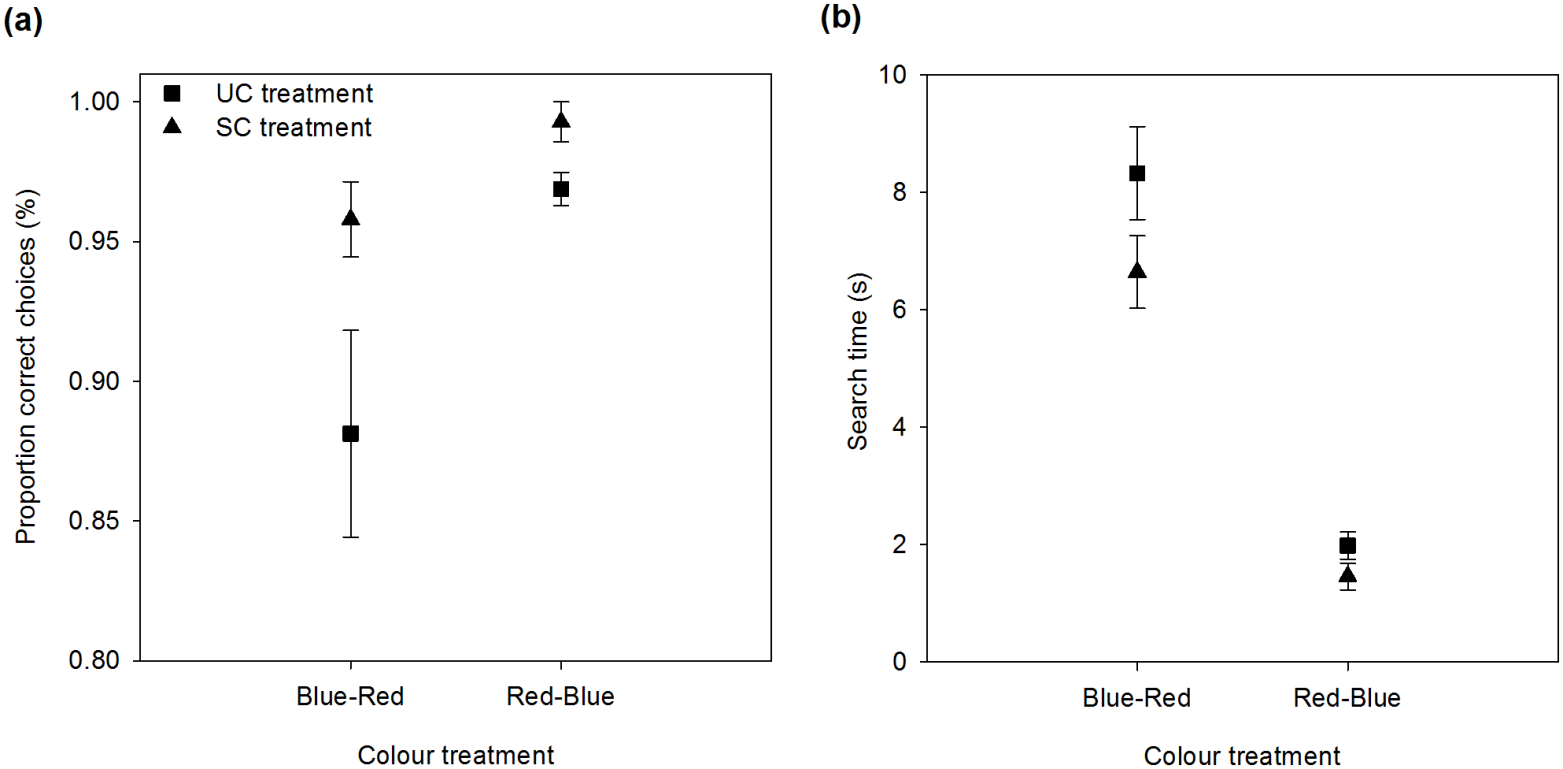
Proportion of correct choices (a) and search time (b) during the novel colour experiment for the unscented (squares) and scented (triangles) odour treatments.

The proportion of correct choices was greater for those bees initially trained with red flowers, searching for novel blue flowers, than for those bees initially trained with blue flowers searching for novel red flowers. Regardless of the colour treatment, the proportion of correct choices increased in the presence of scent (Fig 6a, Table 5). Search time was also greater when bees trained with blue flowers had to search for novel red flowers, decreasing the time in the presence of scent (Fig 6b, Table 5).

**Table 5:** GLM (correct choices) and LM (search time) models with hypothesis-testing for the novel-colour analyses.

Model	Variables			
Correct Choices		X^2^	d.f.	P-value
	OT	10.43	1	**0.001**
	CT	19.16	1	**<0.0001**
Search Time		SS/F	d.f.	P-value
	OT	0.74/5.56	1	**0.02**
	CT	19.62/147.20	1	**<0.0001**

OT = odour treatment, CT = colour treatment

## Discussion

Insects rely upon different sensory information for their daily activities, being highly adaptive regarding their innate and learned preferences, and strongly modulated by the local environmental conditions. From all the variables considered in this study, colour strongly influenced bumblebee behaviour during the foraging activity in many ways. Despite the general idea that pollinators’ visual system and floral signals work synergistically to increase the detection of stimuli and constancy of visits, generalist pollinators seem to overcome situations where this relationship does not seem to be the rule. Red coloration has been pointed out as a strategy to avoid visitation of illegitimate visitors by means of sensorial exclusion. The strategy works only to some degree: bees do visit (UV-absorbing) red flowers in natural communities. The performance of bumblebees searching for our UV-absorbing red flowers improved with training, especially in the absence of odour, which in turn had a positive effect on performance even in the absence of training. Flower size had little effect on bee behaviour. Only its interaction with flower colour had a significant impact on search time. Given that we had only two replicates of each size-round combination, however, it is possible that a weak effect of flower size was masked by the effect of training, and that such effect could be revealed by increasing sample size. Bees searching for red flowers maintained or even reduced their flight speed as training progressed, adjusting their behaviour to minimize the risk of missing flowers, while bees searching for blue flowers increased the flight speed with training. Despite these speed differences, path length was greater when bees searched for red flowers than when they searched for blue flowers, although this difference decreased in the presence of scent. Our scented treatment consisted of only one scent combined with two colours and different sizes. Thus, although the presence of scent affected the response of bumblebees, we cannot conclude that any scent will have the same effect, and the generality of the results remains to be evaluated.

### The exploitation of floral signals in a visually complex background

Background complexity has recently gained attention in the context of foraging dynamics of bumblebees when detecting salient and inconspicuous stimuli [4,9,17]. Bumblebee colour preference changes depending on the salience of stimuli against complex and simple backgrounds. Forrest and Thomson [4] demonstrated that when blue and red UV-absorbing flowers were simultaneously presented against a homogeneous background, bumblebees indistinctly visited both colours, but when a complex background (digital image of natural foliage) was used, bumblebees strongly preferred blue flowers, the most conspicuous stimulus [4]. When the two colours are tested again in a three-dimensional environment, simulating the foliage disposition on nature, in comparison with a simple homogenous background, a similar result is obtained, suggesting that independent of the source, background complexity (two—foliage picture—and three-dimensional presentations) is comparable in the challenge they represent to bees [9]. A different result was obtained by Gegear and colleagues [17] when presenting violet and red artificial flowers in a complex visual background similar to those of Forrest and Thomson [4]: bumblebees showed no colour preference. We did not test bumblebee preference of colours presented simultaneously, but how bumblebees modulated their behaviour in response to different traits (including colour) when foraging in a visually complex background. Our bumblebees searching for red flowers improved accuracy and reduced search time after 10 foraging bouts, but on average were less successful than bees searching for blue flowers. At the end of the last round (22 bouts), bees’ performance was still increasing, indicating that, with training, the exploitation of visually difficult tasks can be overcome. Once the initial sensory, and possibly associated morphological, barriers of exploitation are trespassed, bees can explore such resources assuming some costs—flight speed and total path length adjustments–if the reward is worth it.

**Fig 7:**
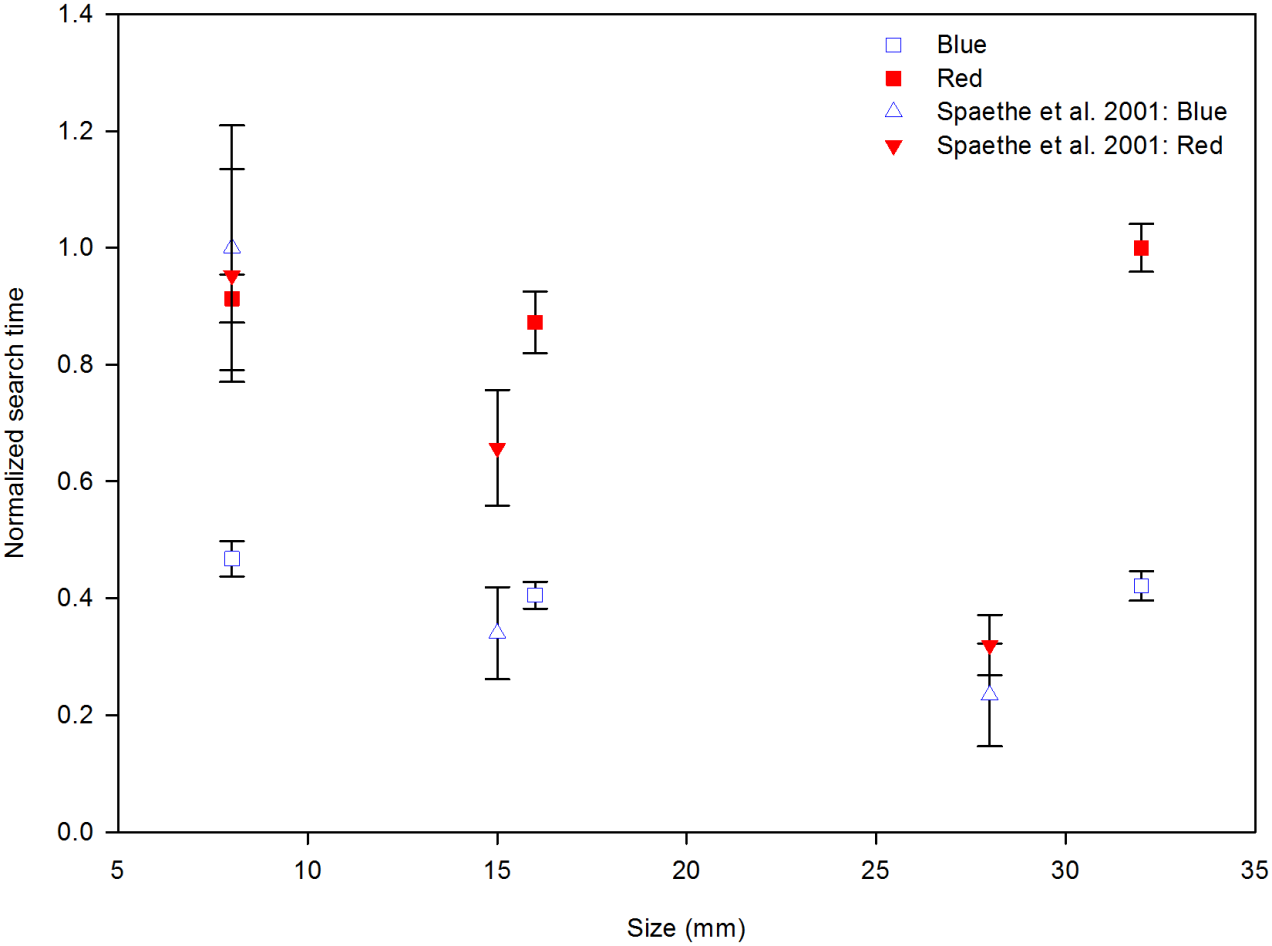
Normalized search times of bumblebees searching for flowers of different sizes and colours in homogeneous (triangles) and complex (squares) backgrounds. For normalization, search times were divided by the maximum search time of their dataset (44.4 s the data reported by Spaethe et al. [3] and 1.22 s for this experiment).

Another floral trait that has been demonstrated to affect the performance of bees during the foraging activity is flower size. In a previous experiment, Spaethe and colleagues [5] demonstrated a significant correlation between search time, colour and size when using a homogeneous green background and odourless flowers. In that study, flowers of three sizes (circles 28, 15, and 8 mm in diameter) were sequentially presented in a descending order, and search time increased as flower size decreased. To make both Spaethe [5] and our data comparable, we normalized search times from both experiments (Fig 7; details in the figure’s legend), ignoring odour treatments—no effect during search time analysis, and general dissimilarities between experiments such as stimulus presentation, bumblebees colonies identity, illumination, and so on. In a homogeneous background, the effect of flower size on search time was stronger than the effect of flower colour. In our setup, however, it was the other way around (Fig 7).

In the previous experiment [5], all bees started searching for large flowers, and then went to medium and small flowers. Search time might have been expected to decrease as the experiment progressed, but it increased: the effect of flower size was strong enough to erase any effect of training in the homogeneous background. Normalized search time differences between experiments could not be explained by the chromatic contrast of stimuli against backgrounds, since these contrasts were similar across experiments (Table 1 of Spaethe et al. [5] and S1 Table).

Gegear and colleagues [17] in a series of manipulations, demonstrated that different trait combinations (such as colour, reward quality/quantity, and flower orientation) work as an integrated functional unit to generate foraging selectivity in bumblebees. When traits, such as colour and flower orientation, were analysed separately, bees did not demonstrate any preference in exploiting each of them, but as soon as the result of trait interaction increased energetical costs, bumblebees expressed avoidance behaviour [17]. In this sense, size could act synergistically together with colour as an important barrier against floral thieves (as those bees visiting red bird-pollinated flowers). In our experimental setup, flower size had little effect on bumblebees’ performance or behaviour. Only search time was affected by the interaction between flower colour and size: bumblebees searching for red flowers took longer to find big flowers (32 mm) than medium (16 mm) and small (8 mm) flowers, while search time was independent of flower size (and tended to decrease as size increased) when flowers were blue.

#### The chromatic—Achromatic information use in flower detection and discrimination

Bees possess two separate but interacting visual pathways for flower detection and discrimination [7,35]. The chromatic pathway is used when targets subtend a large visual angle, while the achromatic pathway, mediated by the green receptors, is used when targets subtend a small visual angle [7,35,36]. Although bees can detect stimuli subtending a large visual angle in the absence of chromatic information, presumably using the achromatic pathway, such detection is difficult [37–39]. This could explain why bumblebees searching for red flowers needed more time to find large than small and medium sized flowers. Another striking point is that bees are very fast at learning tasks based on chromatic contrasts, while they require extended learning to perform tasks based on achromatic contrasts [40]. Our results agree with this observation. In our experiments, bumblebees exploiting red flowers improved their performance with training, while the performance of bees exploiting blue flowers was excellent from the beginning.

#### Visual and olfactory modulation during the foraging activity

Different studies have reported the existence of a trade-off between speed and accuracy [39,41–44], demonstrating that bees adjust their behaviour to the difficulty of the task. In our flight speed experiment, bees searching for blue flowers increased their flight speed as they became more experienced–either because the task became easier or because they learnt that they could increase their speed without making more errors. Bees searching for red flowers, however, kept the same flight speed throughout the experimental sessions. The presence of a second sensory modality (odour) or the combination of multicomponent information (size-colour) did not produce any effect on the flight speed of bees visiting red and blue flowers, suggesting that initial detection of stimuli during the flight, is mainly controlled by the visual input related to the contrast produced by the flower and its background. On the analyses of path length, bumblebees exploiting red flowers travelled shorter distances in the presence of scent. It remains unclear whether bumblebees visiting red flowers used scent to guide landing when approaching flowers [45–47], or as a long-distance cue [23,48]. Independently of the sequence of events, bumblebees integrated signals from different sensory modalities during their foraging activity.

Accuracy was positively affected by the presence of a second sensory cue when detection of stimulus was visually constrained. Bird-pollinated flowers are often characterized as odourless [49]. Nonetheless, flower visitors can make use of flower volatiles, even when they are not directly associated to attraction [50,51]. As for the rest of sensory cues, flower-emitted compounds may trigger both innate behavioural responses and be involved in associative learning processes [11,52,53]. Our results demonstrate that multimodal cues were not advantageous for bees searching for conspicuous blue flowers when against a visually complex background, resulting in redundant information. Redundant information will elicit the same response as information presented through a single modality [24]. Besides, the role of multimodal information can be of relevance for bumblebees during a novel task, where only one of the familiar signals is available, conferring advantage to the experienced bee.

The presence of visual and olfactory traits has been associated to floral constancy in bumblebees, improving decision making by influencing the speed and the accuracy of the decision process [54]. Bumblebees exhibit higher flower constancy when flowers differ in both colour and scent than when flowers differ in colour alone, also learning to choose the complex rewarding flowers faster than those flowers that differed only in the visual modality [54,55]. For the former, the explanation falls in the memory capacity of bumblebees to effectively search for and/or remember multiple combinations of floral traits at the same time, keeping a single flower type in active memory and thereby staying constant on that species during foraging [55,56]. For the choice speed, the salience of complex stimulus might be the answer, since a bimodal stimulus may be detected more quickly than a unimodal stimulus [57]. Under our experimental setup, search time was not positively affected by the complexity of stimulus, but the salience of colours against the background. If search time was determined by salience, blue and scented stimulus might have had a reduced search time in comparison with the other colour-odour combinations.

However, differently from a laboratory controlled situation, bumblebees must face difficult foraging tasks when they must choose amongst dozens of species usually emitting multiple signals and presenting different rewards. How do Hymenoptera floral visitors innately respond to complex floral traits is still an open question, especially when combining multimodal signals [58–61]. They must be able to prioritize the combination of relevant traits in the ecological context they are, or even select some of them to rely on during the foraging activity, ignoring the presence of others, if it comes at some–unnecessary—costs (like memory) [62].

### Novel-colour learning behaviour

When bees form elemental associations between a reward, scent, and colour, these cross-modal relationships are linked in memory [57] and apparently used during new information acquisition. During the novel task experiment, bumblebees trained with blue and red flowers in the presence of odour had a high proportion of correct choices and spent less time searching for the novel flowers than those bees trained with the same colours but in the absence of odour (Fig 6). The presence of scent by itself helped bumblebees to find the novel stimulus in both colour treatments.

Linalool, the most representative floral volatile compound found in our scent, occurs widely in many diurnal flowers pollinated by bees, acting as a appetitive signal and also eliciting innate responses in honeybees [63–66]. Nevertheless, considering the most abundant volatiles in our scent (S2 Table), they comprehend some of the widespread floral volatiles, occurring in more than 50% of angiosperm families, also being some of the 12 most common volatile compounds present in floral scents [67]. The role of such volatiles in bee attraction has been demonstrated by previous studies [61,64,68,69].

Because bees found it easier to find blue than red flowers, bees trained with red flowers and searching for blue flowers during the novel task experiment performed better, being faster and more accurate, easily switching from red to the novel blue flowers (Red-Blue treatment) than bees facing the opposite transition. This behaviour was consistent between odour treatments. When given the opportunity, bumblebees are going to prefer the colour that allows for a better balance between speed and accuracy.

## Concluding remarks

Perception of a stimulus is affected by the contrast it produces against the background. For instance, considering a visually noisy environment, conspicuous and inconspicuous flowers, bumblebee performance was differently affected by the presence of complex floral signals (colour and odour), given that they can explore these signals and use them as cues to find food resources at close and long distances. Whether and how investment in complex floral displays directly or indirectly affects floral visitors, is not fully understood [17,54,70], since most studies focus only on single sensory modalities as an approach towards understanding the role of pollinator cognition on predicting foraging behaviour.

Floral odour is important when the visual task is difficult, as with our UV-absorbing red flowers, or as it might be for the UV-reflecting white flowers naturally visited by bumblebees [13,18]. Multimodal stimuli allow pollinators to use different sensory channels when foraging in different contexts [71]. Bumblebees, as generalist flower visitors, benefit from their capacity of using one or more sensory modalities to improve target detection, when relying on a single sensory modality is inefficient. They adjust their behaviour to facilitate target detection and discrimination, and they might make use of all available sensory inputs and neural pathways, as long as foraging activity can be enhanced without highly energetic costs.

## Supporting information

S1 Fig **(a) Overview of the EPS panel and flight cage.** (b) Detail of platform with red (32 mm) and blue (8 mm) stimuli.
(DOCX)

S1 Table Quantum catches, chromatic and achromatic properties of stimuli. Colour contrast against the average background (CCB), according to the colour hexagon (CH, [1], the colour opponent coding (COC, [2] and the receptor-limited [3] models and achromatic green (GC) and brightness (BG) contrasts, calculated as specified by Spaethe and colleagues [4].
(DOC)

S2 Table Volatiles organic compounds and relative concentrations (%) identified in the lavender (*Lavandula officinalis*) essential oil used as scent. The information was provided by the commercial seller MARNYS ^®^, MARTÍNEZ NIETO, S.A., Health Food and Natural Beauty Laboratory, Cartagena, Spain. Main compounds and concentrations found by the company are comparable to those previously found elsewhere [1].
(DOCX)

## References

[1] Leonard AS, Papaj DR, Dornhaus A. Why are floral signals complex? An outline of functional hypotheses In: Patiny S, editor. Evolution of Plant-Pollinator Relationships. Cambridge: Cambridge University Press; 2011 pp. 261–282.

[2] Rohde K, Papiorek S, Lunau K. Bumblebees (*Bombus terrestris*) and honeybees (*Apis mellifera*) prefer similar colours of higher spectral purity over trained colours. J Comp Physiol A. SPRINGER; 2013;199: 197–210. doi: 10.1007/s00359-012-0783-5 23224278 10.1007/s00359-012-0783-5

[3] Katzenberger TD, Lunau K, Junker RR. Salience of multimodal flower cues manipulates initial responses and facilitates learning performance of bumblebees. Behav Ecol Sociobiol. 2013;67: 1587–1599. doi: 10.1007/s00265-013-1570-1

[4] Forrest J, Thomson JD. Background complexity affects colour preference in bumblebees. Naturwissenschaften. 2009;96: 921–5. doi: 10.1007/s00114-009-0549-2 19444425 10.1007/s00114-009-0549-2

[5] Spaethe J, Tautz J, Chittka L. Visual constraints in foraging bumblebees: flower size and color affect search time and flight behavior. Proc Natl Acad Sci U S A. 2001;98: 3898–903. doi: 10.1073/pnas.071053098 11259668 10.1073/pnas.071053098 PMC31150

[6] Lehrer M, Horridge G a., Zhang SW, Gadagkar R. Shape Vision in Bees: Innate Preference for Flower-Like Patterns. Philos Trans R Soc B Biol Sci. 1995;347: 123–137. doi: 10.1098/rstb.1995.0017

[7] Giurfa M, Vorobyev M, Kevan PG, Menzel R. Detection of coloured stimuli by honeybees: minimum visual angles and receptor specific contrasts. J Comp Physiol A. 1996;178: 699–709.

[8] Ne’eman G, Ne’eman R. Factors determining visual detection distance to real flowers by Bumble bees. J Pollinat Ecol. 2017;20: 1–12.

[9] Rivest SA, Austen EJ, Forrest JRK. Foliage affects colour preference in bumblebees (*Bombus impatiens*): a test in a three-dimensional artificial environment. Evol Ecol. Springer International Publishing; 2017; 1–12. doi: 10.1007/s10682-017-9893-4

[10] Bukovac Z, Dorin A, Finke V, Shrestha M, Garcia J, Avarguès-Weber A, et al Assessing the ecological significance of bee visual detection and colour discrimination on the evolution of flower colours. Evol Ecol. 2016; 1–20. doi: 10.1007/s10682-016-9843-6

[11] Wright GA, Schiestl FP. The evolution of floral scent: the influence of olfactory learning by insect pollinators on the honest signalling of floral rewards. Funct Ecol. 2009;23: 841–851. doi: 10.1111/j.1365-2435.2009.01627.x

[12] Martínez-Harms J, Palacios a G, Márquez N, Estay P, Arroyo MTK, Mpodozis J. Can red flowers be conspicuous to bees? *Bombus dahlbomii* and South American temperate forest flowers as a case in point. J Exp Biol. 2010;213: 564–71. doi: 10.1242/jeb.037622 20118307 10.1242/jeb.037622

[13] Lunau K, Papiorek S, Eltz T, Sazima M. Avoidance of achromatic colours by bees provides a private niche for hummingbirds. J Exp Biol. 2011;214: 1607–1612. doi: 10.1242/jeb.052688 21490268 10.1242/jeb.052688

[14] Dafni A, Bernhardt P, Shmida A, Ivri BY, Greenbaum S, Losito L. Red bowl-shaped flowers : convergence for beetle pollination in the Mediterranean region. Isr J Bot. 1990;39: 81–92.

[15] Rodríguez-Gironés MA, Santamaría L. Why are so many bird flowers red? PLoS Biol. 2004;2: e350 doi: 10.1371/journal.pbio.0020350 15486585 10.1371/journal.pbio.0020350 PMC521733

[16] Bergamo PJ, Rech AR, Brito VLG, Sazima M. Flower colour and visitation rates of Costus arabicus support the “bee avoidance” hypothesis for red-reflecting hummingbird-pollinated flowers. Funct Ecol. 2016;30: 710–720. doi: 10.1111/1365-2435.12537

[17] Gegear RJ, Burns R, Swoboda-Bhattarai KA. “Hummingbird” floral traits interact synergistically to discourage visitation by bumblebee foragers. Ecology. 2017;98: 489–499. doi: 10.1002/ecy.1661 27864943 10.1002/ecy.1661

[18] Chittka L, Waser NM. Why red flowers are not invisible to bees. Isr J Plant Sci. 1997;45: 169–183.

[19] Peitsch D, Fietz A, Hertel H, de Souza J, Ventura DF, Menzel R. The spectral input systems of hymenopteran insects and their receptor-based colour vision. J Comp Physiol A. 1992;170: 23–40. Available: http://www.ncbi.nlm.nih.gov/pubmed/1573568 1573568 10.1007/BF00190398

[20] Chittka L, Shmida A, Troje N, Menzel R. Ultraviolet as a component of flower reflections, and the colour perception of Hymenoptera. Vision Res. 1994;34: 1489–508. Available: http://www.ncbi.nlm.nih.gov/pubmed/8023461 8023461 10.1016/0042-6989(94)90151-1

[21] Skorupski P, Döring TF, Chittka L. Photoreceptor spectral sensitivity in island and mainland populations of the bumblebee, *Bombus terrestris*. J Comp Physiol A. 2007;193: 485–94. doi: 10.1007/s00359-006-0206-6 17333207 10.1007/s00359-006-0206-6

[22] Galizia CG, Kunze J, Gumbert A, Borg-Karlson AK, Sachse S, Markl C, et al Relationship of visual and olfactory signal parameters in a food-deceptive flower mimicry system. Behav Ecol. 2005;16: 159–168. doi: 10.1093/beheco/arh147

[23] Kunze J, Gumbert A. The combined effect of color and odor on flower choice behavior of bumble bees in flower mimicry systems. Behav Ecol. 2001;12: 447–456.

[24] Sánchez D, Nieh JC, Vandame R. Visual and chemical cues provide redundant information in the multimodal recruitment system of the stingless bee *Scaptotrigona mexicana* (Apidae, Meliponini). Insectes Soc. 2011;58: 575–579. doi: 10.1007/s00040-011-0181-y

[25] Chittka L, Spaethe J. Visual search and the importance of time in complex decision making by bees. Arthropod Plant Interact. 2007;1: 37–44. doi: 10.1007/s11829-007-9001-8

[26] Skorupski P, Spaethe J, Chittka L. Visual search and decision making in bees: time, speed, and accuracy. Int J Comp Psychol. 2006;19: 342–357.

[27] Backhaus W. Color opponent coding in the visual system of the honeybee. Vision Res. 1991;31: 1381–1397. 1891826 10.1016/0042-6989(91)90059-e

[28] Chittka L. The colour hexagon: a chromaticity diagram based on photoreceptor excitations as a generalized representation of colour opponency. J Comp Physiol A. 1992;170: 533–543. doi: 10.1007/BF00199331

[29] Vorobyev M, Osorio D. Receptor noise as a determinant of colour thresholds. Proc Biol Sci. 1998;265: 351–8. doi: 10.1098/rspb.1998.0302 9523436 10.1098/rspb.1998.0302 PMC1688899

[30] Vorobyev M, Brandt R, Peitsch D, Laughlin SB, Menzel R. Colour thresholds and receptor noise: behaviour and physiology compared. Vision Res. 2001;41: 639–53. Available: http://www.ncbi.nlm.nih.gov/pubmed/11226508 11226508 10.1016/s0042-6989(00)00288-1

[31] Zuur AF, Ieno EN, Walker NJ, Saveliev AA, Smith GM. Mixed effects models and extensions in ecology with R. New York: Springer New York; 2009 doi: 10.1007/978-0-387-87458-6

[32] Akaike H. Information theory and an extension of the maximum likelihood principle In: Petrov BN, Csaki F, editors. Proceedings of the Second International Symposium on Information Theory. Budapest: Akademiai Kiado; 1973 pp. 267–281.

[33] R Core Team. R: A language and environment for statistical computing [Internet]. Vienna, Austria: R Foundation for Statistical Computing; 2013 http://www.r-project.org/

[34] Bates D, Maechler M. lme4: linear mixed-effects models using Eigen and S4 [Internet]. 2013. http://cran.r-project.org/package=lme4

[35] Giurfa M, Vorobyev M, Brandt R, Posner BB, Menzel R. Discrimination of coloured stimuli by honeybees: alternative use of achromatic and chromatic signals. J Comp Physiol A. 1997;180: 235–243. doi: 10.1007/s003590050044

[36] Dyer AG, Spaethe J, Prack S. Comparative psychophysics of bumblebee and honeybee colour discrimination and object detection. J Comp Physiol A. 2008;194: 617–27. doi: 10.1007/s00359-008-0335-1 18437390 10.1007/s00359-008-0335-1

[37] Hempel de Ibarra N, Vorobyev M, Brandt R, Giurfa M. Detection of bright and dim colours by honeybees. J Exp Biol. 2000;203: 3289–98. Available: http://www.ncbi.nlm.nih.gov/pubmed/11023849 11023849 10.1242/jeb.203.21.3289

[38] Reisenman CE, Giurfa M. Chromatic and achromatic stimulus discrimination of long wavelength (red) visual stimuli by the honeybee *Apis mellifera*. Arthropod Plant Interact. 2008;2: 137–146.

[39] Telles FJ, Rodríguez-Gironés MA. Insect vision models under scrutiny: what bumblebees (*Bombus terrestris terrestris* L.) can still tell us. Sci Nat. 2015;102: 1–13. doi: 10.1007/s00114-014-1256-1 25613579 10.1007/s00114-014-1256-1

[40] Giurfa M, Vorobyev M. The angular range of achromatic target detection by honey bees. J Comp Physiol A. 1998;183: 101–110. doi: 10.1007/s003590050238

[41] Chittka L, Dyer AG, Block F, Dornhaus A. Bees trade off foraging speed accuracy. Nature. 2003;424: 388. 12879057 10.1038/424388a

[42] Chittka L, Skorupski P, Raine NE. Speed-accuracy tradeoffs in animal decision making. Trends Ecol Evol. 2009;24: 400–407. doi: 10.1016/j.tree.2009.02.010 19409649 10.1016/j.tree.2009.02.010

[43] Dyer AG, Chittka L. Bumblebees (*Bombus terrestris*) sacrifice foraging speed to solve difficult colour discrimination tasks. J Comp Physiol A. 2004;190: 759–63. doi: 10.1007/s00359-004-0547-y 15316731 10.1007/s00359-004-0547-y

[44] Rodríguez-Gironés MA, Trillo A, Corcobado G. Long term effects of aversive reinforcement on colour discrimination learning in free-flying bumblebees. PLoS One. 2013;8: e71551 doi: 10.1371/journal.pone.0071551 23951186 10.1371/journal.pone.0071551 PMC3741178

[45] Lunau K. Innate flower recognition in bumblebees (*Bombus terrestris*, *B*. *lucorum*; Apidae): optical signals from stamens as landing reaction releasers. Ethology. 1991;88: 203–214.

[46] Lunau K. Innate recognition of flowers by bumble bees: orientation of antennae to visual stamen signals. Can J Zool. 1992;70: 2139–2144. doi: 10.1139/z92-288

[47] Dobson HEM, Danielson EM, Van Wesep ID. Pollen odor chemicals as modulators of bumble bee foraging on *Rosa rugosa* Thunb. (Rosaceae). Plant Species Biol. 1999;14: 153–166.

[48] Vereecken NJ, Schiestl FP. On the roles of colour and scent in a specialized floral mimicry system. Ann Bot. 2009;104: 1077–1084. doi: 10.1093/aob/mcp208 19692390 10.1093/aob/mcp208 PMC2766200

[49] Cronk Q, Ojeda I. Bird-pollinated flowers in an evolutionary and molecular context. J Exp Bot. 2008;59: 715–727. doi: 10.1093/jxb/ern009 18326865 10.1093/jxb/ern009

[50] Ballantyne G, Willmer P. Nectar Theft and Floral Ant-Repellence: A Link between Nectar Volume and Ant-Repellent Traits? PLoS One. 2012;7: 1–10. doi: 10.1371/journal.pone.0043869 22952793 10.1371/journal.pone.0043869 PMC3430612

[51] Farré-Armengol G, Filella I, Llusia J, Peñuelas J. Floral volatile organic compounds: Between attraction and deterrence of visitors under global change. Perspect Plant Ecol Evol Syst. 2013;15: 56–67. doi: 10.1016/j.ppees.2012.12.002

[52] Carlsson MA, Hansson BS. Detection and Coding of Flower Volatiles in Nectar-Foraging Insects In: Pichersky E, Dudareva N, editors. Biology of Floral Scent. CRC Press; 2006 pp. 243–261. doi: 10.1201/9781420004007.ch11

[53] Wright GA, Baker DD, Palmer MJ, Stabler D, Mustard JA, Power EF, et al Caffeine in floral nectar enhances a pollinator’s memory of reward. Science. 2013;339: 1202–1204. doi: 10.1126/science.1228806 23471406 10.1126/science.1228806 PMC4521368

[54] Kulahci IG, Dornhaus A, Papaj DR. Multimodal signals enhance decision making in foraging bumble-bees. Proc Biol Sci. 2008;275: 797–802. doi: 10.1098/rspb.2007.1176 18198150 10.1098/rspb.2007.1176 PMC2596894

[55] Gegear RJ, Laverty TM. Flower constancy in bumblebees: a test of the trait variability hypothesis. Anim Behav. 2005;69: 939–949. doi: 10.1016/j.anbehav.2004.06.029

[56] Junker RR, Parachnowitsch AL. Working Towards a Holistic View on Flower Traits—How Floral Scents Mediate Plant–Animal Interactions in Concert with Other Floral Characters. J Indian Inst Sci A Multidiscip Rev J. 2015;95: 1–13.

[57] Leonard AS, Masek P. Multisensory integration of colors and scents: insights from bees and flowers. J Comp Physiol A Neuroethol Sensory, Neural, Behav Physiol. 2014;200: 463–474. doi: 10.1007/s00359-014-0904-4 24710696 10.1007/s00359-014-0904-4

[58] Goodale E, Kim E, Nabors A, Henrichon S, Nieh JC. The innate responses of bumble bees to flower patterns: separating the nectar guide from the nectary changes bee movements and search time. Naturwissenschaften. 2014;101: 523–6. doi: 10.1007/s00114-014-1188-9 24879351 10.1007/s00114-014-1188-9

[59] Orbán LL, Plowright CMS. Getting to the start line: how bumblebees and honeybees are visually guided towards their first floral contact. Insectes Soc. 2014;61: 325–336. doi: 10.1007/s00040-014-0366-2 25328168 10.1007/s00040-014-0366-2 PMC4196025

[60] Lunau K, Fieselmann G, Heuschen B, van de Loo A. Visual targeting of components of floral colour patterns in flower-naïve bumblebees (*Bombus terrestris*; Apidae). Naturwissenschaften. 2006;93: 325–8. doi: 10.1007/s00114-006-0105-2 16568268 10.1007/s00114-006-0105-2

[61] Knauer AC, Schiestl FP. Bees use honest floral signals as indicators of reward when visiting flowers. Ecol Lett. 2015;18: 135–143. doi: 10.1111/ele.12386 25491788 10.1111/ele.12386

[62] Odell E, Raguso RA, Jones KN. Bumblebee foraging responses to variation in floral scent and color in Snapdragons (*Antirrhinum*: Scrophulariaceae). Am Midl Nat. 1999;142: 257–265. doi: 10.1674/0003-0031(1999)142[0257:BFRTVI]2.0.CO;2

[63] Raguso RA, Pichersky E. A day in the life of a linalool molecule: Chemical communication in a plant-pollinator system. Part 1: Linalool biosynthesis in ßowering plants. Plant Species Biol. 1999;14: 95–120.

[64] Garbuzov M, Ratnieks FLW. Quantifying variation among garden plants in attractiveness to bees and other flower-visiting insects. Funct Ecol. 2014;28: 364–374. doi: 10.1111/1365-2435.12178

[65] Nouvian M, Hotier L, Claudianos C, Giurfa M, Reinhard J. Appetitive floral odours prevent aggression in honeybees. Nat Commun. Nature Publishing Group; 2015;6: 1–10. doi: 10.1038/ncomms10247 26694599 10.1038/ncomms10247 PMC4703898

[66] Dötterl S, Vereecken NJ. The chemical ecology and evolution of bee–flower interactions: a review and perspectives. Can J Zool. 2010;88: 668–697. doi: 10.1139/Z10-031

[67] Knudsen JT, Eriksson R, Gershenzon J, Ståhl B. Diversity and Distribution of Floral Scent. Bot Rev. 2006;72: 1–120. doi: 10.1663/0006-8101(2006)72[1:DADOFS]2.0.CO;2

[68] Parachnowitsch AL, Raguso RA, Kessler A. Phenotypic selection to increase floral scent emission, but not flower size or colour in bee-pollinated *Penstemon digitalis*. New Phytol. 2012;195: 667–675. doi: 10.1111/j.1469-8137.2012.04188.x 22646058 10.1111/j.1469-8137.2012.04188.x

[69] Byers KJRP, Bradshaw HD, Riffell JA. Three floral volatiles contribute to differential pollinator attraction in monkeyflowers (*Mimulus*). J Exp Biol. 2014;217: 614–623. doi: 10.1242/jeb.092213 24198269 10.1242/jeb.092213 PMC3922836

[70] Goulson D, Cruise JL, Sparrow KR, Harris AJ, Park KJ, Tinsley MC, et al Choosing rewarding flowers; perceptual limitations and innate preferences influence decision making in bumblebees and honeybees. Behav Ecol Sociobiol. 2007;61: 1523–1529. doi: 10.1007/s00265-007-0384-4

[71] Goyret J, Kelber A, Pfaff M, Raguso R a. Flexible responses to visual and olfactory stimuli by foraging *Manduca sexta*: larval nutrition affects adult behaviour. Proc R Soc London, B. 2009;276: 2739–45. doi: 10.1098/rspb.2009.0456 19419987 10.1098/rspb.2009.0456 PMC2839956

