# Supplementary material for: Multimodal cues provide redundant information for bumblebees when the stimulus is visually salient, but facilitate red target detection in a naturalistic background
Source: PLoS One. 2017 Sep 12;12(9):e0184760. doi: 10.1371/journal.pone.0184760 (PMC5595325; doi:10.1371/journal.pone.0184760)

**Supplementary data 1**

**S1 Fig. (a) Overview of the EPS panel and flight cage**. (b) Detail of platform with red (32 mm) and blue (8 mm) stimuli.


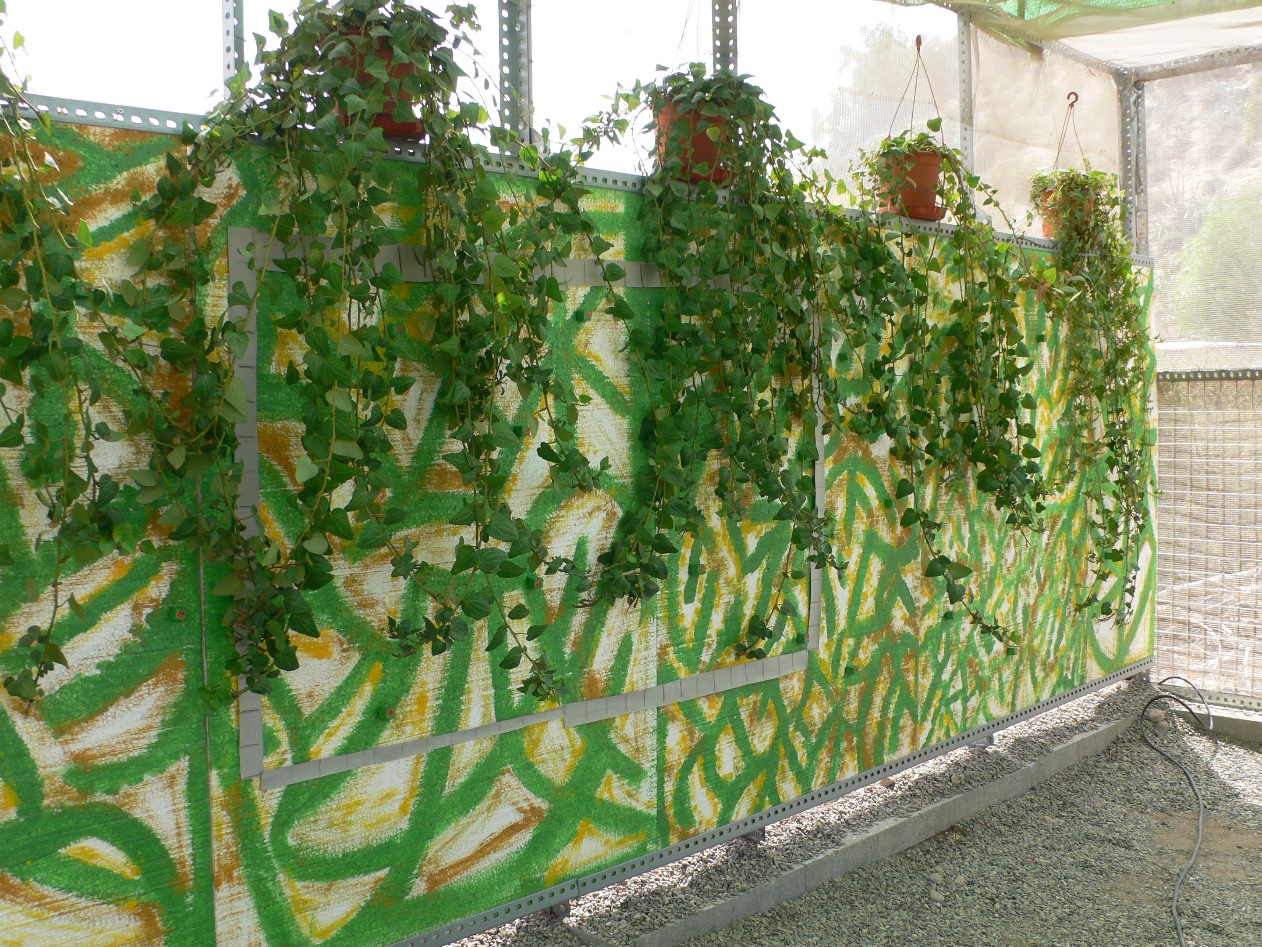
**(a)**


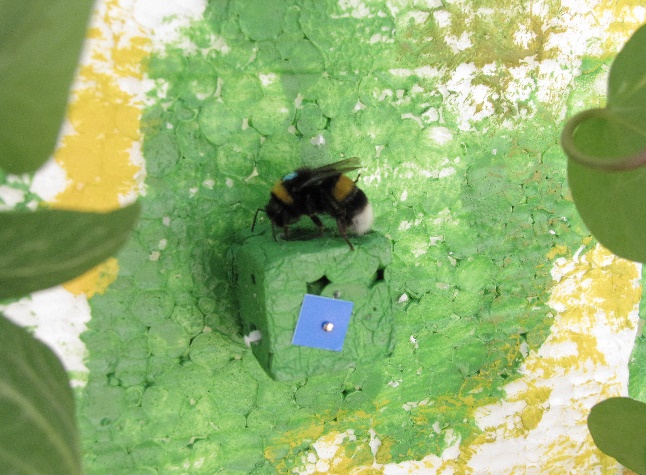
(b)


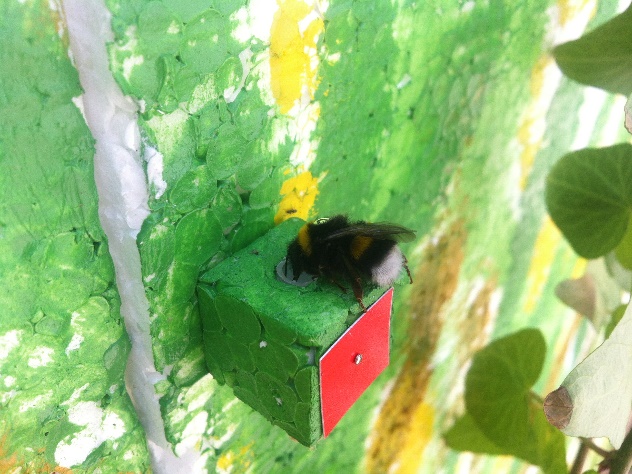

Supplement: S1 Fig — (a) Overview of the EPS panel and flight cage. (b) Detail of platform with red (32 mm) and blue (8 mm) stimuli. (DOCX) [file pone.0184760.s001.docx]
