# Supplementary material for: Multimodal cues provide redundant information for bumblebees when the stimulus is visually salient, but facilitate red target detection in a naturalistic background
Source: PLoS One. 2017 Sep 12;12(9):e0184760. doi: 10.1371/journal.pone.0184760 (PMC5595325; doi:10.1371/journal.pone.0184760)
Supplement: S1 Table — Colour contrast against the average background (CCB), according to the colour hexagon (CH, [1], the colour opponent coding (COC, [2] and the receptor-limited [3] models and achromatic green (GC) and brightness (BG) contrasts, calculated as specified by Spaethe and colleagues [4]. (DOC) [file pone.0184760.s002.doc]

**Supplementary data 2**

**S1 Table. Quantum catches, chromatic and achromatic properties of stimuli.** Colour contrast against the average background (CCB), according to the colour hexagon (CH, [1], the colour opponent coding (COC, [2] and the receptor-limited [3] models and achromatic green (GC) and brightness (BG) contrasts, calculated as specified by Spaethe and colleagues [4].

|  | **Quantum catches** | | | **CCB** | | | **GC** | **BC** |
| --- | --- | --- | --- | --- | --- | --- | --- | --- |
| **Stimulus** | **UV** | **Blue** | **Green** | **CH** | **COC** | **RN** |  |  |
| Blue | 0.93 | 2.19 | 0.91 | 0.21 | 5.78 | 0.56 | -0.02 | 0.14 |
| Red | 0.05 | 0.03 | 0.10 | 0.05 | 0.94 | 0.07 | -0.41 | -1.33 |

**References**

1. Chittka L. The colour hexagon: a chromaticity diagram based on photoreceptor excitations as a generalized representation of colour opponency. J Comp Physiol A. 1992;170: 533–543. doi:10.1007/BF00199331

2. Backhaus W. Color opponent coding in the visual system of the honeybee. Vision Res. 1991;31: 1381–1397.

3. Vorobyev M, Osorio D. Receptor noise as a determinant of colour thresholds. Proc Biol Sci. 1998;265: 351–8. doi:10.1098/rspb.1998.0302

4. Spaethe J, Tautz J, Chittka L. Visual constraints in foraging bumblebees: flower size and color affect search time and flight behavior. Proc Natl Acad Sci U S A. 2001;98: 3898–903. doi:10.1073/pnas.071053098
