# Supplementary material for: Multimodal cues provide redundant information for bumblebees when the stimulus is visually salient, but facilitate red target detection in a naturalistic background
Source: PLoS One. 2017 Sep 12;12(9):e0184760. doi: 10.1371/journal.pone.0184760 (PMC5595325; doi:10.1371/journal.pone.0184760)
Supplement: S2 Table — The information was provided by the commercial seller MARNYS ®, MARTÍNEZ NIETO, S.A., Health Food and Natural Beauty Laboratory, Cartagena, Spain. Main compounds and concentrations found by the company are comparable to those previously found elsewhere [1]. (DOCX) [file pone.0184760.s003.docx]

**Supplementary data 3**

**S2 Table. Volatiles organic compounds and relative concentrations (%) identified in the lavender (*Lavandula officinalis*) essential oil used as scent.** The information was provided by the commercial seller MARNYS ®, MARTÍNEZ NIETO, S.A., Health Food and Natural Beauty Laboratory, Cartagena, Spain. Main compounds and concentrations found by the company are comparable to those previously found elsewhere [1].

| **Compound name** | **Percentages (%)** |
| --- | --- |
| Eucalyptol | 1.30 |
| Cis-ocimene | 3.31 |
| Trans-ocimene | 2.45 |
| 3-Octanone | 1.49 |
| Linalool | 32.39 |
| Linalyl acetate | 30.18 |
| Caryophyllene | 4.21 |
| Terpinen-4-ol | 3.36 |
| Lavandulyl acetate | 3.12 |
| Trans-beta-Farnesene | 4.29 |
| Lavandulol | 1.51 |
| Terpineol | 1.51 |

1. Marín I, Sayas-Barberá E, Viuda-Martos M, Navarro C, Sendra E. Chemical Composition, Antioxidant and Antimicrobial Activity of Essential Oils from Organic Fennel, Parsley, and Lavender from Spain. Foods. 2016;5: 1–10. doi:10.3390/foods5010018
